# Exploring Piezo1, Piezo2, and TMEM150C in human brain tissues and their correlation with brain biomechanical characteristics

**DOI:** 10.1186/s13041-023-01071-5

**Published:** 2023-12-20

**Authors:** Arjun Raha, Yuning Wu, Lily Zhong, Jatheeshan Raveenthiran, Minji Hong, Aftab Taiyab, Li Wang, Bill Wang, Fei Geng

**Affiliations:** 1 School of Biomedical Engineering, McMaster University, Hamilton, ON Canada; 2 Integrated Biomedical Engineering and Health Sciences Program, McMaster University, Hamilton, ON Canada; 3 Department of Pathology and Molecular Medicine, McMaster University, Hamilton, ON Canada; 4 Department of Anesthesia, McMaster University, Hamilton, ON Canada; 5 Department of Surgery, McMaster University, Hamilton, ON Canada; 6 W Booth School of Engineering Practice and Technology, McMaster University, Hamilton, ON Canada

**Keywords:** Brain biomechanics, Cellular mechanotransduction, Piezo1, Piezo2, TMEM150C, Viscoelastic properties, YAP/β-catenin axis

## Abstract

Unraveling the intricate relationship between mechanical factors and brain activity is a pivotal endeavor, yet the underlying mechanistic model of signaling pathways in brain mechanotransduction remains enigmatic. To bridge this gap, we introduced an in situ multi-scale platform, through which we delineate comprehensive brain biomechanical traits in white matter (WM), grey-white matter junctions (GW junction), and the pons across human brain tissue from four distinct donors. We investigate the three-dimensional expression patterns of Piezo1, Piezo2, and TMEM150C, while also examining their associated histological features and mechanotransduction signaling networks, particularly focusing on the YAP/β-catenin axis. Our results showed that the biomechanical characteristics (including stiffness, spring term, and equilibrium stress) associated with Piezo1 vary depending on the specific region. Moving beyond Piezo1, our result demonstrated the significant positive correlations between Piezo2 expression and stiffness in the WM. Meanwhile, the expression of Piezo2 and TMEM150C was shown to be correlated to viscoelastic properties in the pons and WM. Given the heterogeneity of brain tissue, we investigated the three-dimensional expression of Piezo1, Piezo2, and TMEM150C. Our results suggested that three mechanosensitive proteins remained consistent across different vertical planes within the tissue sections. Our findings not only establish Piezo1, Piezo2, and TMEM150C as pivotal mechanosensors that regulate the region-specific mechanotransduction activities but also unveil the paradigm connecting brain mechanical properties and mechanotransduction activities and the variations between individuals.

**Supplementary Information:**

The online version contains supplementary material available at 10.1186/s13041-023-01071-5.

## Introduction

The human brain, an intricate nexus of neuronal networks, remains one of the most enigmatic frontiers in biomedical research. The interplay between mechanical forces and brain activity is a puzzle and unlocking this puzzle holds the promise of transformative insights into brain function, with profound implications for our understanding of neurological disorders and the development of precision medicine [1, 2].

The intricate interplay between mechanical forces and brain function has emerged as a critical avenue of investigation, underpinning the understanding of how the central nervous system senses and responds to its mechanical microenvironment [3]. Despite the undeniable significance of mechanical cues in regulating brain activity, a comprehensive mechanistic framework elucidating the signaling pathways involved in brain mechanotransduction has remained elusive. This gap in knowledge hinders our ability to decipher the intricate relationship between mechanical factors and neuronal processes, thereby limiting our understanding of brain health and disease [1].

One of the essential components of the brain, the white matter (WM), represents areas primarily composed of myelinated axonal fibers [4]. These fibers form the communication pathways between different regions of grey matter (GM) [2, 4, 5]. Traditionally, WM has been associated with the transmission of electrical signals. However, recent research has shed light on its pivotal role in mechanotransduction [6]. The mechanical properties of WM, such as its elasticity and stiffness, can affect the transmission and propagation of mechanical signals within the brain [7].

At the junction of GM (neuronal cell bodies) and WM (axonal fibers), known as the grey-white matter junction (GW junction), crucial neurological processes unfold [4]. This region plays a crucial role in information processing and signal transmission within the brain [4]. It is a site of complex interactions between different types of cells, including neurons, glial cells, and the local vasculature [2, 4, 5]. Mechanotransduction processes at the GW junction are of particular interest because they can influence the integration and propagation of mechanical signals across different brain regions [8].

The pons, another integral part of the brain, acts as a bridge connecting various regions, including the cerebral cortex, cerebellum, and spinal cord [4, 9]. Within the pons lie numerous fiber tracts that serve as essential conduits for transmitting both electrical and mechanical signals between different parts of the brain [9]. Moreover, the pons plays a multifaceted role in various vital functions, such as sleep regulation [10], respiratory control [11], and motor coordination [12].

Previously we have demonstrated the stiffness responsiveness via YAP mechanotransduction [13] and mechanosensitive channel Piezo1 in a YAP-dependent manner [13–16]. These prior investigations underscore our investigation into unraveling the intricate relationships between mechanical cues, cellular signaling pathways, and the brain’s structural and functional attributes.

In the present research, we conducted a comprehensive investigation into these cerebral domains, including the GW junction, the pons, and WM. Within these cerebral landscapes, we unveil the presence of three pivotal mechanosensors—Piezo1, Piezo2, and TMEM150C—entities that have hitherto occupied a relatively obscure niche within the domain of neurobiology. Specifically, we cast the spotlight upon the YAP/β-catenin axis, a pivotal signaling pathway that orchestrates cellular responses to mechanical stimuli. Through this investigation, we establish compelling correlations among the expression profiles of these mechanosensitive entities, the mechanical attributes characterizing cerebral regions, and the region-specific mechanotransduction phenomena. In presenting this multidimensional framework, our research offers not only a holistic understanding of brain biomechanics but also a unified paradigm poised to unveil the mysteries underpinning the brain’s responsiveness to mechanical forces, and thereby catalyze a transformation in our comprehension of cerebral function and dysfunction.

## Materials and methods

### Tissue preparation

Fresh Human brain specimens were taken from donors. Once the donor passes away, their bodies are transferred to the McMaster anatomy lab within 4 h, and the whole brain is removed using standard neuropathology techniques under sterile conditions. The contralateral hemisphere (area unaffected by the tumor) was used as representative non-cancerous tissue. The following regions were sampled from each brain: cortex, basal ganglia, thalamus, caudate head, pons, cerebellum, cervical spinal cord, and corpus callosum. Some areas of the cortex were further sub-sampled, including grey matter, white matter, and the grey-white matter junction. All tissues were placed into ice-cold PBS and collected within 8 h postmortem. Tissue sections from each brain region were compressed using the MicroTester system (CellScale Biomaterials Testing). Immunoblotting analysis was performed using capillary electrophoresis (ProteinSimple - Abby) as per manufacturer guidelines. Immunofluorescent staining was performed as previously described [17].

### Mechanical testing

All mechanical testing was performed within 24 h postmortem. Mechanical testing was performed using the Microtester from CellScale Biomaterial Testing. The apparatus uses a piezo-electric actuator (0.1$\mu$m resolution) to compress samples with force resolution down to 10nN. The tester also uses a high-resolution CCD imaging (1536 × 1536 pixels and 5 Hz data rate) camera to track the displacement of the sample and cantilever beam deflection throughout the testing procedure. Appropriate cantilever beam diameters were selected to achieve the greatest force resolution (0.2032 mm cantilever). In preparation, a 3 mm biopsy punch was used to extract cylindrical cores of the brain that were 3 mm in diameter and 2 mm in height. The tissue cores were loaded onto the testing stage and submerged in a PBS bath. The sample was positioned under a 6 mm x 6 mm metal platen which was attached to the cantilever beam. The platen and cantilever were lowered to contact the brain specimen, ensuring that the platen contact was flush against the sample. Using the CCD camera, the height, and average sample width were measured and recorded in the software SquisherJoy (CellScale Biomaterial Testing).

Two types of compression tests were performed on the various brain regions: quasi-static compression and stepwise-ramp compression. In quasi-static compression, the tissue was slowly compressed up to 10% strain over ten minutes. Stepwise ramp compressions consisted of cyclic loading of the tissues at 2.5%, 5%, 7.5%, and 10% strain. The compression profile is as follows: (1) 20 s of compression up to a given strain, (2) 10 s of “hold” period where the platen remains at a constant displacement while the average force is measured, and (3) 20 s of recovery period where the sample is de-compressed. Young’s modulus was obtained by plotting the stress versus strain curve in MATLAB and taking the slope of the elastic region (2.5% compression) for quasi-static compressions. The relaxation modulus was obtained by curve fitting the region of stress relaxation from the 7.5% stepwise compression. A Zener model was used to describe the stress relaxation behavior of the tissue using the following equation: ${\sigma }_{1}={\epsilon }_{7.5\%}*\left(a{e}^{-bt} \right) + {\sigma }_{e}$ where ${\sigma }_{1}$ represented the stress (in Pa) at time t and ${\epsilon }_{7.5\%}$ was a constant representing the percent strain during the hold phase. The model was solved for $a$, $b$ and ${{\sigma }}_{e}$, which represent the spring term (Pa), decay term (1/t), and equilibrium stress value (Pa) at 7.5% strain, respectively. The data was analyzed in GraphPad prisms utilizing a student’s t-test and one-way ANOVA test to assess the differences between mechanical data of different brain regions.

### Immunofluorescent staining

All tissue slides were deparaffinized by washing them in a series of 100% xylene and ethanol gradients (100%, 95%, and 70%) before a final rinse in distilled water. Antigen retrieval was performed by incubating sections in 10mM sodium citrate at 85–90 °C for 20 min. Following this, the sections were allowed to cool at room temperature while being submerged in the hot sodium citrate for another 20 min. The sections were then blocked using 5% normal donkey serum (NDS) in 1X PBS for 1 h at room temperature in a humidified chamber. Primary Piezo1 antibody (Novus Biologicals, NBP1-78537) was diluted 1:50 in 1X PBS before applying to tissue and incubating at 4 °C overnight. Sections were then washed twice using 1X PBS before applying AlexaFluro 568 donkey anti-rabbit IgG (H + L) secondary (Thermo Fisher Scientific A10042) to 1:200 in 1.5% NDS and allowed to incubate for 1 h at room temperature. The sections were then washed three times in 0.1%PBST with a tinfoil cover to prevent photobleaching. Finally, 30-40$\mu$l of ProLong Gold mounting media with DAPI stain (Thermo Fisher Scientific P36931) was applied, after which the slides were stored at 4 °C in preparation for imaging. All images were taken under Zeiss Axio Observer Z1 and were analyzed using Fiji based on the area fraction of intensity of positive staining. Piezo1 positive cells were identified using a custom algorithm in Fiji. Briefly, relative fluorescence units (RFU) were obtained through cell segmentation analysis. Initially, cells were segmented based on the nuclei counterstain by applying a colour threshold for the blue-green channels. The watershed function was used to separate touching nuclei as some cells were close to one another. Using a particle size of 200, a mask was generated that counted the number of nuclei and signal intensity of DAPI (nuclei stain) for each cell. Next, the Voronoi mask was applied to isolate cell boundaries and further segment images based on the staining patterns of each mechanotransduction protein (Piezo1, Piezo2, and TMEM150C). Similar to nuclei measurements, both a count and signal intensity were measured for the number of positively stained cells. Both masks (nuclei mask and Voronoi mask) were overlayed to isolate the regions of interest (RIO) using matching indices for the nuclei and respective mechanotransduction markers. RFU values were then calculated by taking the ratio of the mechanotransduction marker signal over the DAPI signal.

### Immunoblotting

Flash-frozen tissue was roughly pulverized using mortar and pestle before adding lysis buffer (RIPA buffer) with protease inhibitor (05892970001, Roche) at a 1:5 ratio (tissue: buffer). The mixture was homogenized for ~ 30 s at medium speed using a tissue homogenizer from Kinematica (cat# CH-6010), after which the contents were centrifuged at 500xg for 8 min at 4 °C. The supernatant was removed and used as input for immunoblotting. Pierce™ BCA Protein Assay Kit (Thermo Scientific, 23,227) and Tecan microplate reader (Infinite M200 Pro, Tecan) were used in combination to quantify the tissue lysate concentration.

The expression levels of YAP, phosphorylated YAP (pYAP), β-catenin, Piezo1, and β-actin (internal control) were determined by an automated immunoblotting system (ProteinSimple, Abby, AY2093), which detects proteins of interest via capillary electrophoresis (CE). The electropherogram is generated through an in-capillary immunoassay. Briefly, proteins are loaded into a matrix and undergo electrophoresis. Proteins are then UV cross-linked to the capillaries and the capillary is subsequently cleared of the matrix. The process continues by probing the capillary with a primary antibody, followed by an HRP-conjugated secondary antibody, and a chemiluminescence substrate [18, 19].

Brain tissue lysates were prepared at 0.5 $\mu g$/$\mu L$ with the use of 0.1x Sample Diluent buffer and 5 x Fluorescent Master Mix, which were provided in the manufacturer’s kits (ProteinSimple, SM-W004, and SM-W007). Next, the sample mixtures were heated at 95 °C for 5 min before loading. The primary antibodies used in this experiment included: Yap (D8H1X) XP® Rabbit mAb (Cell Signaling, 14,074 S, 1:50), Phospho-YAP (Ser127) Antibody (Cell Signaling, 4911 S, 1:50), β-Catenin (D10A8) XP® Rabbit mAb (Cell Signaling, 8480 S, 1:250), PIEZO1 Antibody - BSA Free (Novus Biologicals, NBP1-78537, 1:20), and Monoclonal Anti-β-Actin antibody produced in mouse (Millipore Sigma, A5441, 1:50). Anti-Rabbit (ProteinSimple, DM-001) and Anti-Mouse secondary antibodies (ProteinSimple, 042–205) were used for detection based on the primary antibodies’ host species. The derived sample mixtures, along with other required reagents, were loaded into the assay plates according to the Abby Loading Protocol provided by the manufacturer. Two assay modules were used in this experiment, the 12–230 kDa and 66-440 kDa Separation Modules (ProteinSimple, Abby kits, SM-W004 and SM-W007). Default assay settings were applied to each run, and the results were evaluated using the Compass for SW Software (ProteinSimple).

### Statistical analysis

**Fig. 1:**
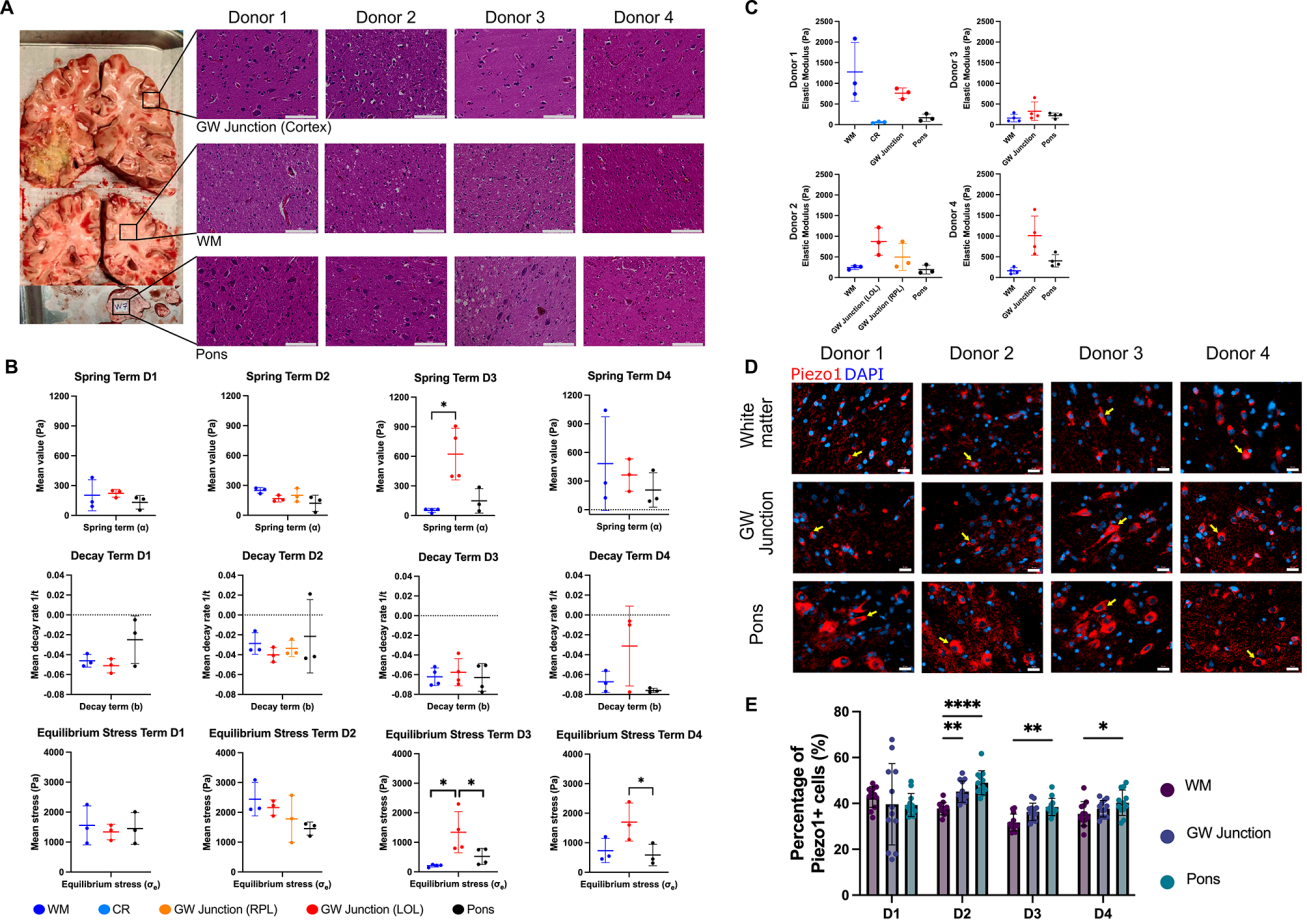
Characterization of brain biomechanics, histological features, and Piezo1 distribution pattern in diverse brain regions from the donors. (**A**) Coronal brain sections and representative H&E images demonstrating regional tissue architecture (scale bar = 100$\mu$m). (**B**) Quantification of viscoelastic properties across different brain locations. Donors are separated based on columns; Donor 1 (D1), Donor 2 (D2), Donor 3 (D3), and Donor 4 (D4); figure legend shows WM, Corona Radiata (CR), GW Junction from Right Parietal Lobe (RPL), GW Junction from Left Occipital Lobe (LOL), and Pons. One-way ANOVA demonstrates significant differences in spring term between the GW junction and white matter of the corona radiata in Donor 3. Similarly, the equilibrium stress of the GW junction was shown to be significantly different compared to the pons and white matter in donor 3. Only comparisons between the GW junction and pons present significant differences in equilibrium stress in donor 4, *p < 0.05, **p < 0.01. (**C**) Individualized comparison of Elastic Modulus (Pa) of different brain regions. (**D**) Representative immunofluorescent staining of brain regions showing Piezo1 (red) and cell nuclei (blue), scale bar = 20$\mu$m. Yellow arrows show examples of Piezo1 positive cells. **(E)** Quantification of the percentage of Piezo1 positive cells based on immunofluorescent (IF) staining presented in Fig. 1D; one-way ANOVA was used showing *p < 0.05, **p < 0.01, ****p < 0.0001. Created with BioRender.com

Correlation analyses between Piezo1 intensity and mechanical parameters, including stiffness, were performed using Stata version 17 (StataCorp version 17). We summarized the Piezo1 relative fluorescent intensity, stiffness, and viscoelastic parameters using median and interquartile range (IQR) due to non-normal data (Fig. 1B-C). Pooled comparisons between Piezo1 and mechanical parameters were performed using Kruskal-Wallis tests. The Bonferroni post hoc tests were used. Correlations between Piezo1, Piezo2, and TMEM150C relative fluorescence versus each mechanical property were quantified using Spearman’s rho. Comparisons were 2-tailed, with a threshold *p*-value of 0.05. Comparisons between viscoelastic parameters were performed using one-way ANOVA tests in GraphPad Prism. Similarly, mechanosensor comparisons and regional relative fluorescent intensities were compared using one-way ANOVA. Comparisons between brain regions and the percentage of mechanosensor-positive cells were compared using two-way ANOVA.

## Results

In this study, we conducted a comprehensive characterization of the mechanical profiles and mechanotransduction proteins within three distinct brain regions: the GW junction of the cortex, WM, and tissue extracted from the pons. The specific sampling locations within the brain are visually indicated in Fig. 1A.

To assess the mechanical behavior of the brain tissue, we examined stress-relaxation phenomena at a 7.5% strain. These data were effectively curve-fitted using a Zener model [2, 20], characterized by its spring term ($\alpha$), decay term (b), and equilibrium stress (${\sigma }_{e}$). This viscoelastic model is described by the equation, ${\sigma }_{t}={\epsilon }_{7.5\%}*\left(a{e}^{-bt} \right) + {\sigma }_{e}$. Our analysis revealed significant variability in both the spring term and equilibrium stress within each brain region, as well as variations across different regions when individual donors were examined (Fig. 1B). Notably, significant differences in equilibrium stress were observed between the GW junction and the corona radiata, as well as between the GW junction and the pons in donor 3 (Fig. 1B). Furthermore, in donor 4, the equilibrium stress between the GW junction and the pons was found to be significantly higher (Fig. 1B).

When data from all donors were aggregated (data not shown), it became evident that the GW junction exhibited a larger spring term and equilibrium stress term compared to the pons, with statistical significance (p < 0.05). Utilizing a quasi-static compression model [20], we generated tissue compression data and subsequently calculated the elastic modulus. Comparative analysis of the elastic modulus at 2.5% strain under quasi-static compression revealed that GW junctions exhibited greater stiffness in comparison to regions such as the pons and WM (Fig. 1C).

**Fig. 2:**
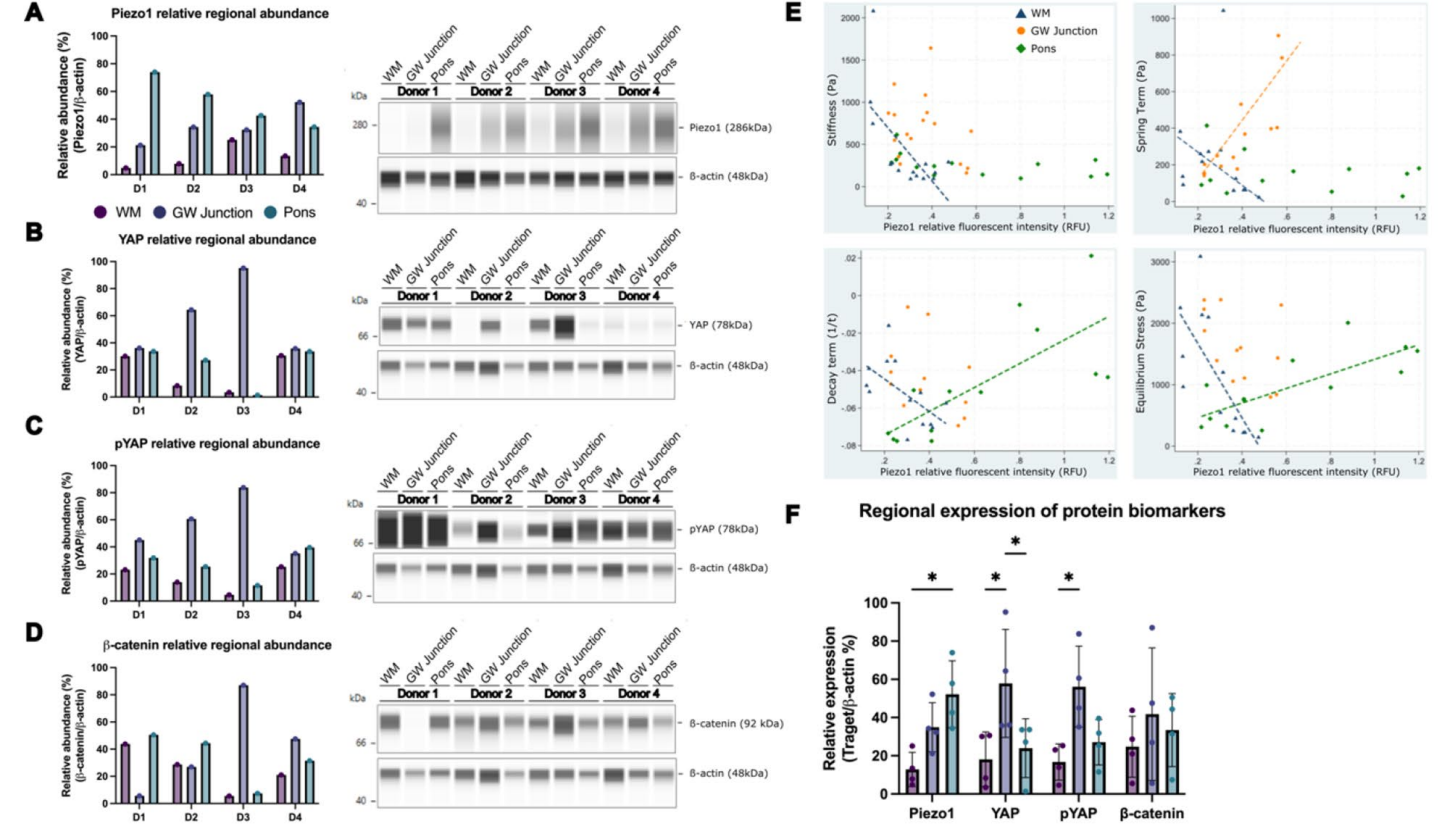
Piezo1 expression profiling, cellular mechanotransduction, and biomechanical correlation analysis across brain regions of different donors. The relative abundance of (**A**) Piezo1, (**B**) YAP, (**C**) pYAP, and (**D**) β-catenin in the regions of WM (magenta), GW junction (purple), and pons (green) for individual donors (D1-4). (**E**, top left) Spearman correlation between Piezo1 relative fluorescence units (RFU) and stiffness; GW Junction (orange) *ns*, WM (blue) r = -0.5341, p < 0.05, pons (green) *ns*. (E, top right) Correlation between Piezo1 regional RFU and spring term ($\alpha$); GW junction r= 0.8791, and WM r = -0.5341, *p* < 0.05. (E, bottom left) Correlation between Piezo1 regional RFU and decay term (b); WM r = -0.6758, and pons r = 0.7692, *p* < 0.05. (E, bottom right) Correlation between Piezo1 regional RFU and equilibrium stress term (${\sigma }_{e})$; WM r = -0.8571, pons r = 0.7198, *p* < 0.05. (**F**) Comparison of protein expressed based on brain region for all donors, *p < 0.05

Given the observed variability in mechanical properties across different brain regions, as depicted in Fig. 1B and C, our study aimed to identify the pivotal mechanosensors characterizing each region. Considering the clinical profiles of the donors, we conducted both pooled and individualized profiling of cellular mechanotransduction markers, including YAP, pYAP (phosphorylated YAP), β-catenin, and the mechanosensitive protein Piezo1. Notably, Piezo1 channels are transmembrane proteins highly expressed in neurons and glia [21, 22]. They play a crucial role in sensing changes in microenvironmental stiffness and transmitting these mechanical cues into intracellular signals [17, 23]. To investigate the distribution of Piezo1-positive cells across different brain regions, we conducted staining and quantification (Fig. 1D and E). Interestingly, while the other brain regions exhibited a relatively consistent range of 40–50% Piezo1-positive cells, the pons displayed a significantly higher proportion in three out of four donors (Figs. 1E and 2F). These consistent patterns of Piezo1 expression across brain regions were further confirmed using capillary electrophoresis and immunoblotting (Fig. 2A).

We explored the expression of Piezo1 downstream effectors, including YAP and its phosphorylated (inactive) isoform pYAP (Fig. 2B and C, and 2F). Notably, GW junction exhibited high YAP expression levels, with relatively high abundance in Donor 3. Moreover, the distribution of β-catenin expression showed unique patterns among regions across different donors and was notably increased in the GW junction of Donor 3 (Fig. 2D). Remarkably, our analysis revealed a significant correlation between Piezo1 expression and the stiffness of WM (Fig. 2E, top left). Additionally, viscoelastic parameters such as spring term displayed a positive correlation with Piezo1 expression levels in the GW junction while the correlation with WM showed a negative correlation (Fig. 2E, top right). The decay phenomenon (parameter ‘b’) and equilibrium stress (${\sigma }_{e})$ (Fig. 2E, bottom left and bottom right respectively) appeared to be significantly correlated with Piezo1 intensity in WM and the pons. Furthermore, donor-specific differences in YAP and pYAP expression have been observed. Thus, the biomechanical characteristics associated with Piezo1 vary depending on the specific region and individual variations among donors.

**Fig. 3:**
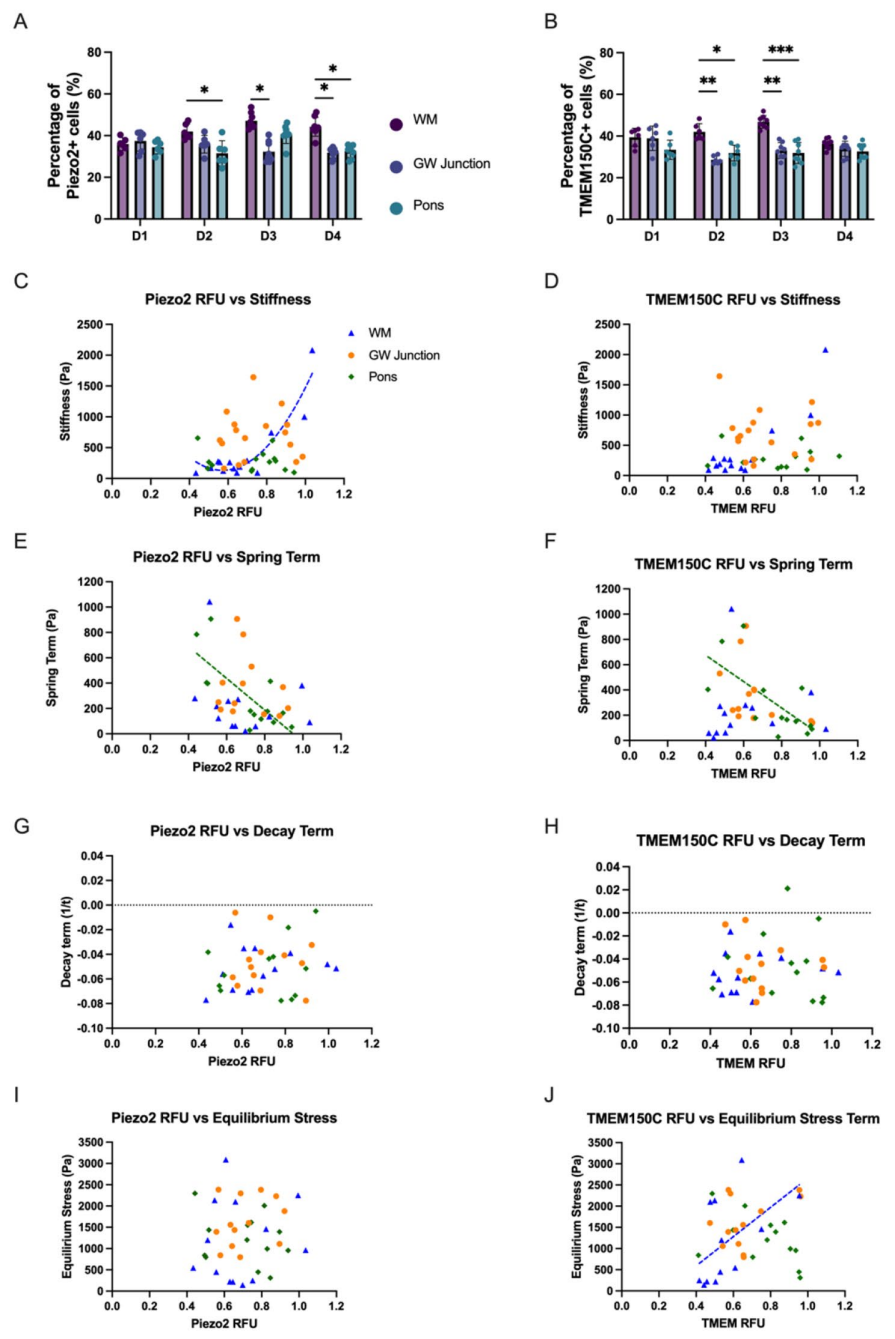
The correlation of Piezo2/TMEM150C relative fluorescence units and percentage of positive cell population to brain mechanical properties. Percentage of Piezo2 positive cells (**A**) and TMEM150C positive cells (**B**) across different brain regions (*p < 0.05, **p < 0.01, ***p < 0.001). Spearman correlation between relative mechanosensor RFU and stiffness for Piezo2 (**C**) and TMEM150C (**D**) shows a significant correlation between Piezo 2 RFU and stiffness in WM only (r = 0.5516, *p* < 0.05). Spearman correlation between mechanosensor RFU and spring term for Piezo2 (**E**) and TMEM150C (**F**) with significant correlation in the Pons for Piezo2 RFU-spring term (r = -0.5602, *p* < 0.05) and TMEM150C RFU-spring term (r = -0.6429, p < 0.05). Spearman correlation between mechanosensor RFU and decay term for Piezo2 (**G**) & TMEM150C (**H**) showing no significant correlations across all brain regions. Spearman correlation between RFU and equilibrium stress for Piezo2 (**I**) and TMEM150C (**J**) where a significant correlation exists between TMEM150C RFU and equilibrium stress in white matter only (r = 0.5604, *p* < 0.05)

To gain a more comprehensive understanding of brain mechanotransduction and identify potential mechanosensors beyond Piezo1, we analyzed the expression profiles of Piezo2 and TMEM150C in three distinct brain regions: WM, GW junction, and the pons (Fig. 3). Similar to Piezo1, we investigated Piezo2 and TMEM150C from both expression level (relative fluorescence units, RFU) and the percentage of positive cell population.

Our results revealed intriguing patterns of expression for both Piezo2 and TMEM150C across these brain regions. In Fig. 3A, we observed diverse expression patterns associated with Piezo2 expression in these regions. Notably, in Donor 1 (D1), there were no significant differences in Piezo2 expression levels between WM, GW junction, and pons regions. This suggests that Piezo2 may fulfill a similar mechanosensory role across these brain regions, indicating a shared mechanotransduction mechanism regulating brain activity. However, in Donor 2 (D2), Donor 3 (D3), and Donor 4 (D4), Piezo2 expression exhibited significant disparities. Specifically, in D2, WM displayed significantly higher Piezo2 expression compared to the pons, while in D3, Piezo2 exhibited higher expression in WM than the GW junction. In D4, the WM region demonstrated higher Piezo2 expression compared to both the GW junction and the pons (Fig. 3A).

The expression profile of TMEM150C, another candidate mechanosensor, presented intriguing findings in Fig. 3B. In D1 and D4, there were no significant differences in TMEM150C expression levels between WM, GW junction, and the pons. In contrast, D2 and D3 displayed a distinct elevation of TMEM150C expression in the WM compared to the GW junction or pons regions (Fig. 3B). This might suggest variations in TMEM150C across these regions that differ among individual donors.

To further explore the relationship between mechanical properties and Piezo2/TMEM150C (both expression level and percentage of positive cell population) across brain regions, we conducted correlation analyses similar to what was done for Piezo1. Notably, we observed significant positive correlations between stiffness and Piezo2 RFU in the WM (r = 0.5516, p < 0.05). Regarding the spring term ($\alpha$), we identified a significant correlation with Piezo2 RFU in the pons (r = -0.5604, p < 0.05) and TMEM150C RFU in the pons (r = -0.6429, p < 0.05). Finally, a correlation was observed between TMEM150C RFU and equilibrium stress in the WM only (r = 0.5604, p < 0.05) (Fig. 3C-J).

**Fig. 4:**
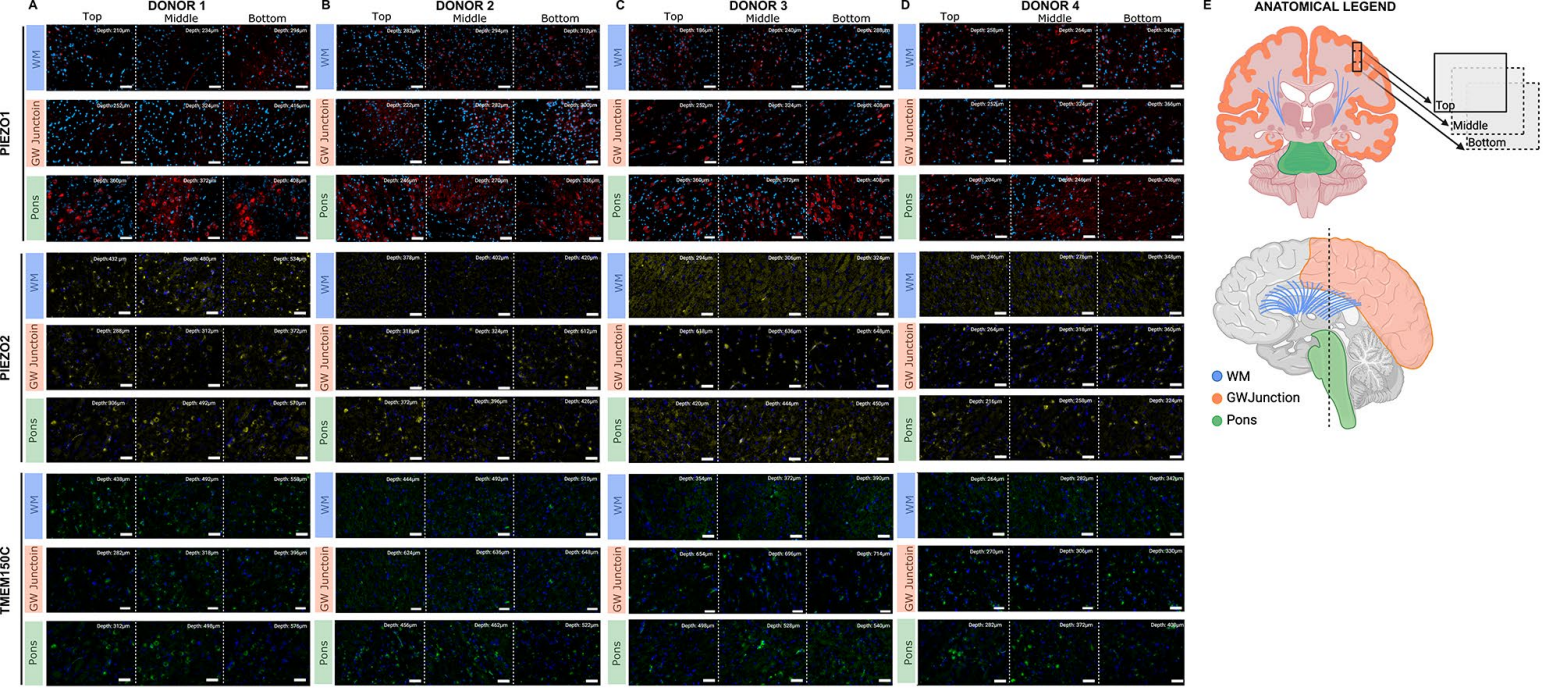
Three-dimensional expression profile of Piezo1, Piezo2, TMEM150C across brain regions (WM, GW Junction, Pons) from four donors. (**A-D**) The top three rows represent the expression profile of Piezo1; the middle three rows represent the expression profile of Piezo2; the bottom three rows represent the expression profile of TMEM150C. (**A-D**) Columns represent the spatial position of each field of view showing the top, middle, and bottom sections of tissue in each brain region. Depth measurements for each field of view are labeled in the top right corner of each image (**A-D**). (**A**) expression analysis of and mechanosensor Piezo1, Piezo2, TMEM150C across brain regions (WM, GW Junction, Pons), (**B**) Donor 2 IF images for each brain region and mechanosensor, (**C**) Donor 3 IF images for each brain region and mechanosensor, (**D**) IF images for each brain region and mechanosensor Donor 4. (**E**) Anatomical legend for each brain region showing a coronal cross section (top) with an example of the spatial arrangement of each tissue layer for a particular brain region; the sagittal section (bottom) shows the spatial arrangement of each brain region. Scale bar = 50$\mu$m

Hence, we identified Piezo1, Piezo2, and TMEM150C as mechanosensors in WM, GW junction and the pons through the correlation analyses linking region-specific expression (expression levels and the percentage of positive cell lines) with corresponding biomechanical characteristics as shown in Fig. 1E (Piezo1), Fig. 3A (Piezo2), and Fig. 3B (TMEM150C). We further sought to explore the three-dimensional localization of Piezo1, Piezo2, and TMEM150C in WM, GW junction, and the pons. An anatomical legend in Fig. 4E provides a visual reference for the location of each tissue region in a coronal view, illustrating the dimensional layering of the tissue sections, followed by a sagittal view illustrating the spatial arrangement of each tissue region. Our results demonstrated that the staining patterns of Piezo1, Piezo2, and TMEM150C remained consistent across different vertical planes within the tissue sections, as shown in Fig. 4A-D.

## Discussion

In this research, we present in vivo data to unravel the intricate relationship between mechanosensors Piezo1, Piezo2, and TMEM150C. We’ve accomplished this by developing an in situ multi-scale platform that integrates region-specific brain tissue mechanics, histological characterization, and cellular mechanotransduction profiling, all conducted across samples obtained from four individual donors. This unique approach has allowed us to create comprehensive brain biomechanical profiles, shedding light on the mechanistic signaling pathways involved in brain mechanotransduction.

One of our significant findings is the establishment of a connection between the mechanosensitive molecule Piezo1, the YAP/β-catenin axis, and brain mechanics, as illustrated in Fig. 2. Through our investigations, we’ve delved into the dynamic brain biomechanical profiles underlying passive relaxation and compression. These profiles are governed by the viscoelastic properties and stiffness of brain tissue, respectively. Our analysis reveals that grey matter and the GW junction are stiffer (approximately 656 Pa) compared to the pons (approximately 255 Pa) and WM (approximately 227 Pa) (p < 0.05). Notably, these mechanical profiles, including viscoelastic properties, cellular mechanotransduction, and the mechanosensor Piezo1, exhibit unique characteristics across individual donors (Figs. 1 and 2).

Piezo1 expression is known to be associated with sensing mechanical cues [24], Interestingly, our data show significantly higher Piezo1 expression in the pons compared to other brain regions, despite the pons being one of the softer regions we examined. This observation suggests that Piezo1 might serve an alternative function in the pons, possibly related to additional mechanical stresses or unique vascular features inherent to the region during development [15, 25].

Beyond Piezo1, we’ve also uncovered intriguing findings. There is a significant positive correlation between TMEM150C RFU and equilibrium stress in the white matter, implying that higher TMEM150C expression levels may be associated with increased equilibrium stress in this brain region. As a potential mechanosensory protein [26, 27], TMEM150C may play a role in sensing and responding to mechanical forces in the white matter, potentially affecting its structural integrity and mechanotransduction-related cellular processes. Additionally, our data suggest that Piezo2 may be functionally relevant as a mechanosensor in the WM, indicating its potential importance in processes like axonal guidance or myelination [28, 29].

The correlations observed between brain mechanical properties and the expression of Piezo1, Piezo2, and TMEM150C suggest the existence of intricate mechanotransduction processes in the brain (Figs. 1 and 3). The unique correlations with stiffness, spring term, and equilibrium stress in the white matter and the pons indicate that these mechanosensors may respond to specific mechanical cues in region-specific manners (Fig. 1). This highlights the complexity of brain mechanotransduction, where different brain regions may have distinct mechanosensory mechanisms that contribute to their unique functions and responses to mechanical stimuli.

The observed staining patterns of mechanosensors (Piezo1, Piezo2, and TMEM150C) at different depths within tissue sections provide valuable insights into the spatial distribution of these mechanosensors (Fig. 4). The consistency of staining patterns across different vertical planes (top, middle, and bottom) suggests that the expression of these biomarkers remains relatively constant throughout the depth of the tissue sections. This uniform distribution within tissue sections indicates the potential for consistent mechanosensory responses across various tissue layers. The anatomical legend presented in Fig. 4 aids in visualizing the spatial orientation of each tissue region in both coronal and sagittal views. This comprehensive visualization helps contextualize the correlation data within the distinct brain regions studied, enhancing our understanding of how mechanical properties and mechanosensor expression are interconnected within the human brain.

**Fig. 5:**
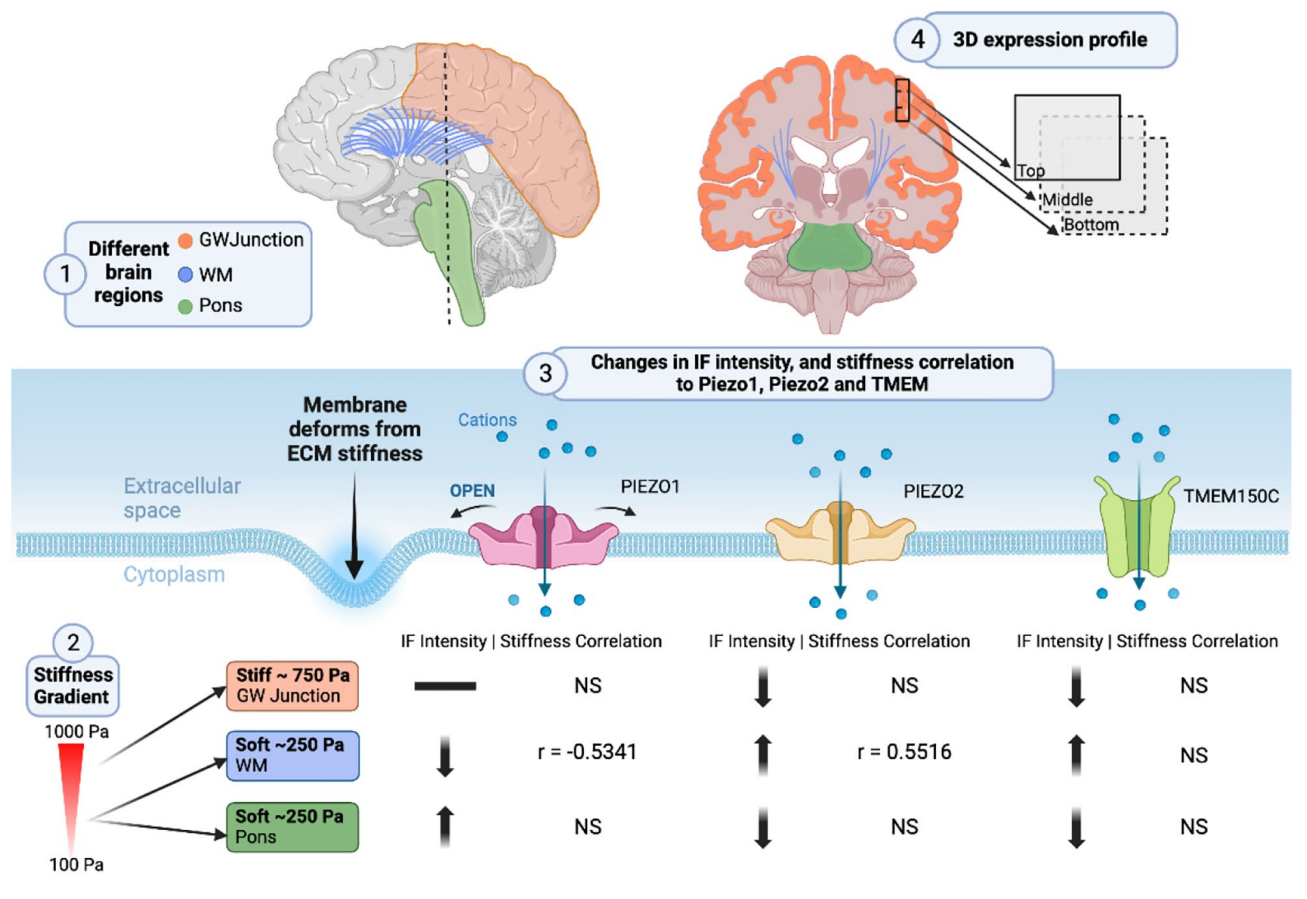
The illustration depicting the complex sensitivity of mechanosensors Piezo1, Piezo2, and TMEM150C to mechanical stimuli within various brain regions (including GW Junction, WM, and Pons) and across three-dimensional space

As shown in Fig. 5, our results unveil the paradigm connecting brain mechanical properties and mechanotransduction activities via the mechanosensors Piezo1, Piezo2, and TMEM150C, in a region-specific manner. This suggests that mechanotransduction plays a pivotal role in the region-specific regulation of brain activity. The mapping of viscoelastic properties and elastic modulus in the GW junction, the pons, and WM provides a comprehensive understanding of the mechanical characteristics of these brain regions. The GW junction, in particular, is known to be a critical area for information processing and communication between different brain regions.

Different regions of the brain have distinct functions and structures. WM, GW junctions, and the pons serve different purposes and have unique cellular compositions [28, 29]. Consequently, the mechanotransduction machinery in these regions may need to be adapted to suit their specific functions. This adaptation can involve variations in the expression levels of mechanosensitive genes. By assessing the correlation between the expression levels of Piezo1, Piezo2, and TMEM150C and the stiffness properties of these brain regions, we have established a link between gene expression and mechanical properties. Understanding these differences in gene expression and mechanical properties may help researchers identify why certain brain disorders affect specific regions or why certain treatments are more effective in one region compared to another. Meanwhile, we also identified the donor-to-donor variability in the absolute expression levels of YAP and pYAP, as highlighted in Fig. 2B and C. This variability could stem from several factors, including individual differences among donors, sample processing, as well as potential technical variations. Further study on donor-to-donor variability could pave the way for more precise, personalized treatments for brain disorders.

In summary, our study highlights compelling correlations between mechanical properties and the expression of mechanosensors across different brain regions. These correlations offer insights into the potential roles of Piezo1, Piezo2, and TMEM150C in responding to tissue stiffness and mechanical stress, respectively. The consistent staining patterns of mechanosensors across tissue depths underscore the robustness of their expression within various layers. Ultimately, these findings contribute to unraveling the intricate mechanotransduction processes in the human brain, advancing our understanding of how mechanical cues influence brain function and disease. Further investigations into the functional implications of these correlations could provide a more comprehensive understanding of mechanobiological mechanisms in neurological contexts.

### Donor information

Donor 1 (MBT413): 58-year-old Male– Right temporal GBM, treated with resection, chemotherapy (temozolomide), and radiotherapy, followed by progression and finally succumbed to disease progression.

Donor 2 (MBT415): 78-year-old Female – Bilateral frontal GBM, never received treatment, deteriorated, and passed away.

Donor 3 (MBT445): 29-year-old Male - Has undergone three resections in 2019, 2020, and 2021 previously treated with temozolomide. Completed treatment with dexamethasone. Received postoperative radiotherapy, Durvalumab, and Olaparib. IDH1 mutation p53 positive.

Donor 4 (MBT 446): 65-year-old Male - MGMT promoter methylation was detected, focally positive for p53, IDH1 wildtype.

### Electronic supplementary material

Below is the link to the electronic supplementary material.

Supplementary Material 1

## Data Availability

Data and materials will be made available based on reasonable request.

## Abbreviations

ANOVA: Analysis of Variance
BSA: Bovine Serum Albumin
CR: Corona Radiata
DAPI: 4′,6-diamidino-2-phenylindole
D1: Donor 1
D2: Donor 2
D3: Donor 3
D4: Donor 4
GW Junction: Grey-White Junction
IF: Immunofluorescent
IQR: Interquartile Range
LOL: Left Occipital Lobe
NBF: Neutral Buffer Formalin
NDS: Normal Donkey Serum
Pa: Pascal
PBS: Phosphate Buffered Saline
pYAP: phosphorylated Yes Associated Protein
PFA: Paraformaldehyde
RFU: Relative fluorescence units
RPL: Right Parietal Lobe
WM: White Matter
YAP: Yes Associated Protein
α: Spring terms
*b*: Decay term
σ_*e*_: Equilibrium stress
ε_7.5%_: 7.5 percent strain
σ_*t*_: Stress at time t
