# Supplementary material for: Exploring Piezo1, Piezo2, and TMEM150C in human brain tissues and their correlation with brain biomechanical characteristics
Source: Mol Brain. 2023 Dec 20;16:83. doi: 10.1186/s13041-023-01071-5 (PMC10731887; doi:10.1186/s13041-023-01071-5)
Supplement: Supplementary file 1 — Supplementary Material 1 [file 13041_2023_1071_MOESM1_ESM.docx]

**Supplemental information**

**Figures**


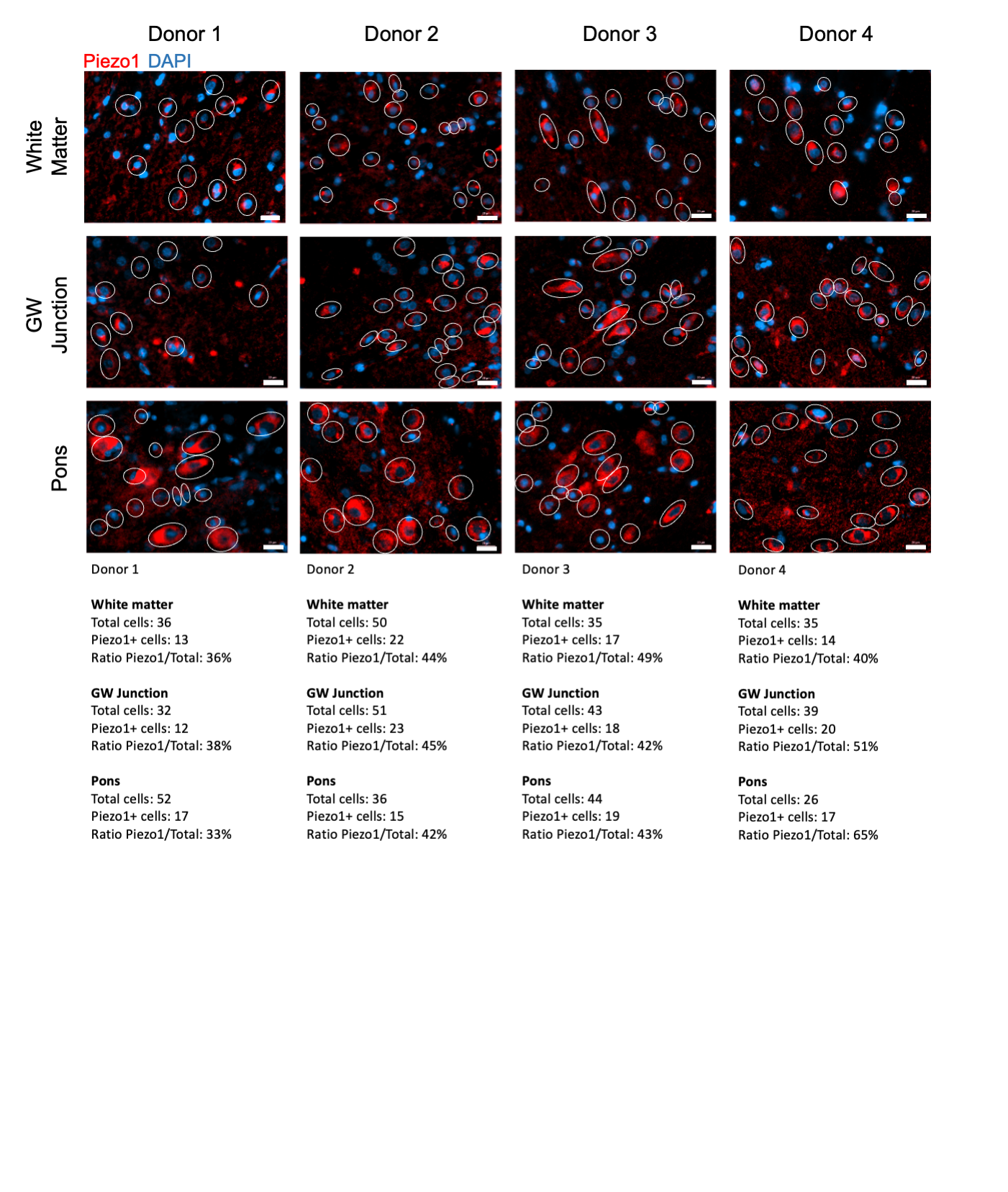


**Fig. S1** Validation of immunofluorescent quantification results from Fig 1D using a representative image gallery. The image gallery shows Piezo1 staining for each brain donor and the respective brain regions including white matter (WM), grey-white junction (GW Junction), and pons. The white circles and ovals represent cells stained positively for Piezo1. Under each image column (representing tissue donors) is a summary displaying the number of total cells, Piezo1 positive (Piezo1+) cells, and the percentage of Piezo1+ cells. Notice that all percentages are ~40% matching the quantification shown in Figure 1D.


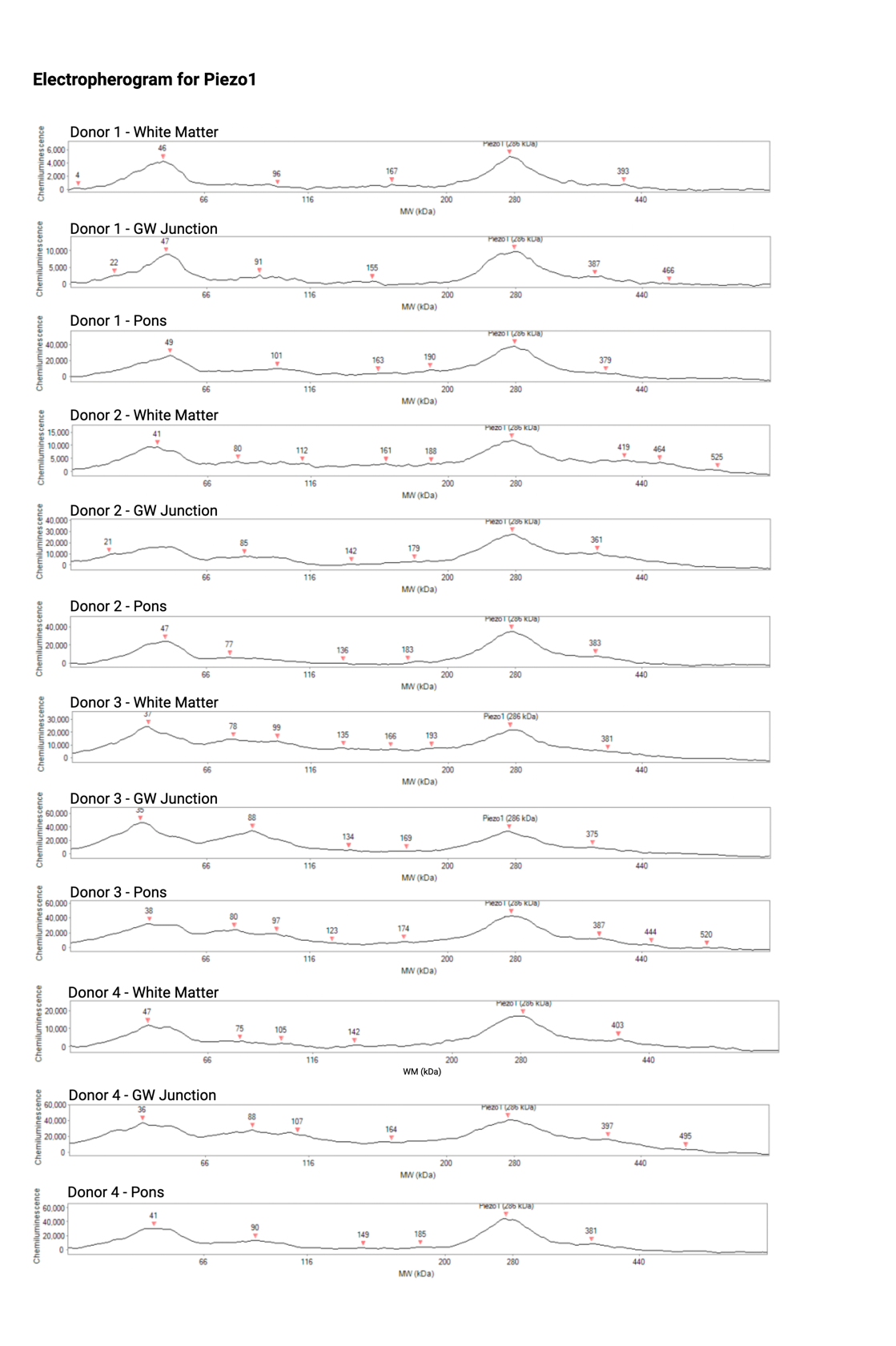

**Fig. S2** Electropherogram of capillary electrophoresis for Piezo1 protein across brain regions (WM, GW Junction, and Pons) from different donors. Electropherograms correspond to Fig. 2A showing chemiluminescence versus molecular weight (MW) indicating a signal of bound proteins to the capillaries in the form of peaks on the graphs. Piezo1 has a molecular weight of 286kDa and is captured as such by its respective antibody on the electropherogram. Each donor and the respective brain regions have a signal peak for Piezo1 at 286kDa.


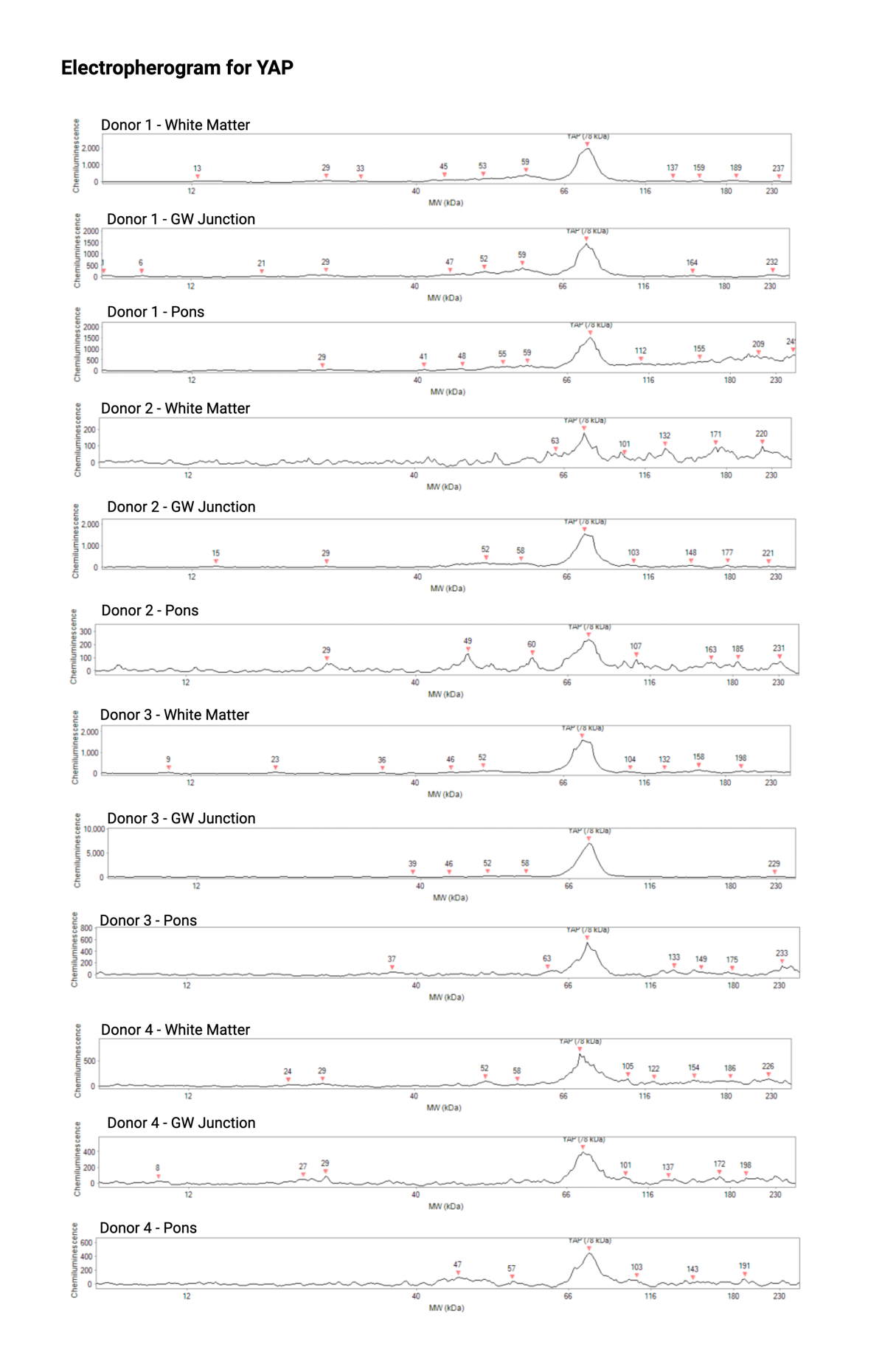

**Fig. S3** Electropherogram of capillary electrophoresis for YAP across brain regions (WM, GW Junction, and Pons) from different donors. Electropherograms corresponding to Fig. 2B showing chemiluminescence versus molecular weight (MW) indicating signal of bound proteins to the capillaries in the form of peaks on the graphs. YAP has a molecular weight of ~78kDa and is captured as such by its respective antibody on the electropherogram. Each donor and the respective brain regions have a signal peak for YAP at 78kDa.

**
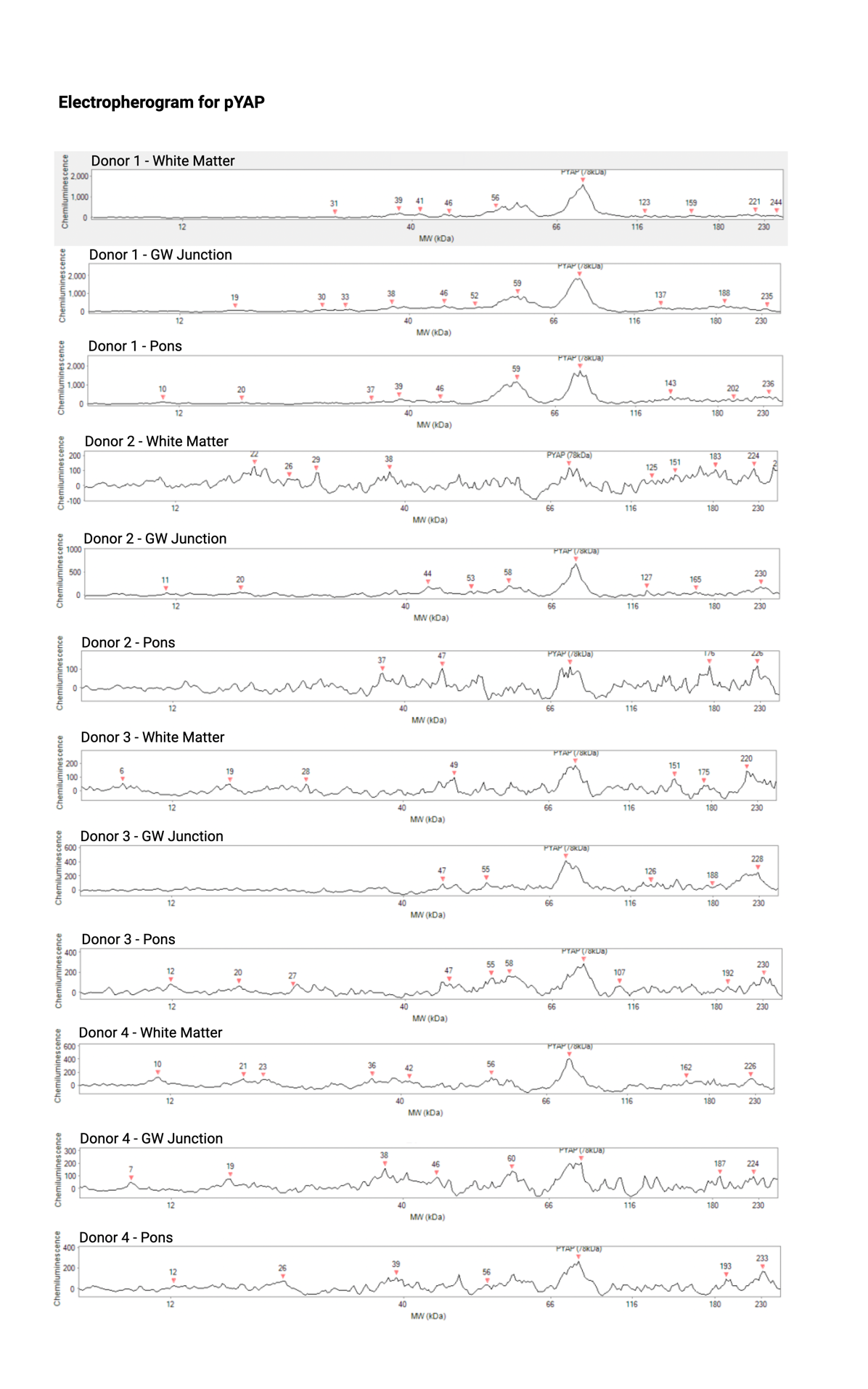
**

**Fig. S4** Electropherogram of capillary electrophoresis for pYAP across brain regions (WM, GW Junction, and Pons) from different donors. Electropherograms corresponding to Fig. 2C showing chemiluminescence versus molecular weight (MW) indicating signal of bound proteins to the capillaries in the form of peaks on the graphs. pYAP has a molecular weight of ~78kDa and is captured as such by its respective antibody on the electropherogram. Each donor and the respective brain regions have a signal peak for YAP at 78kDa.


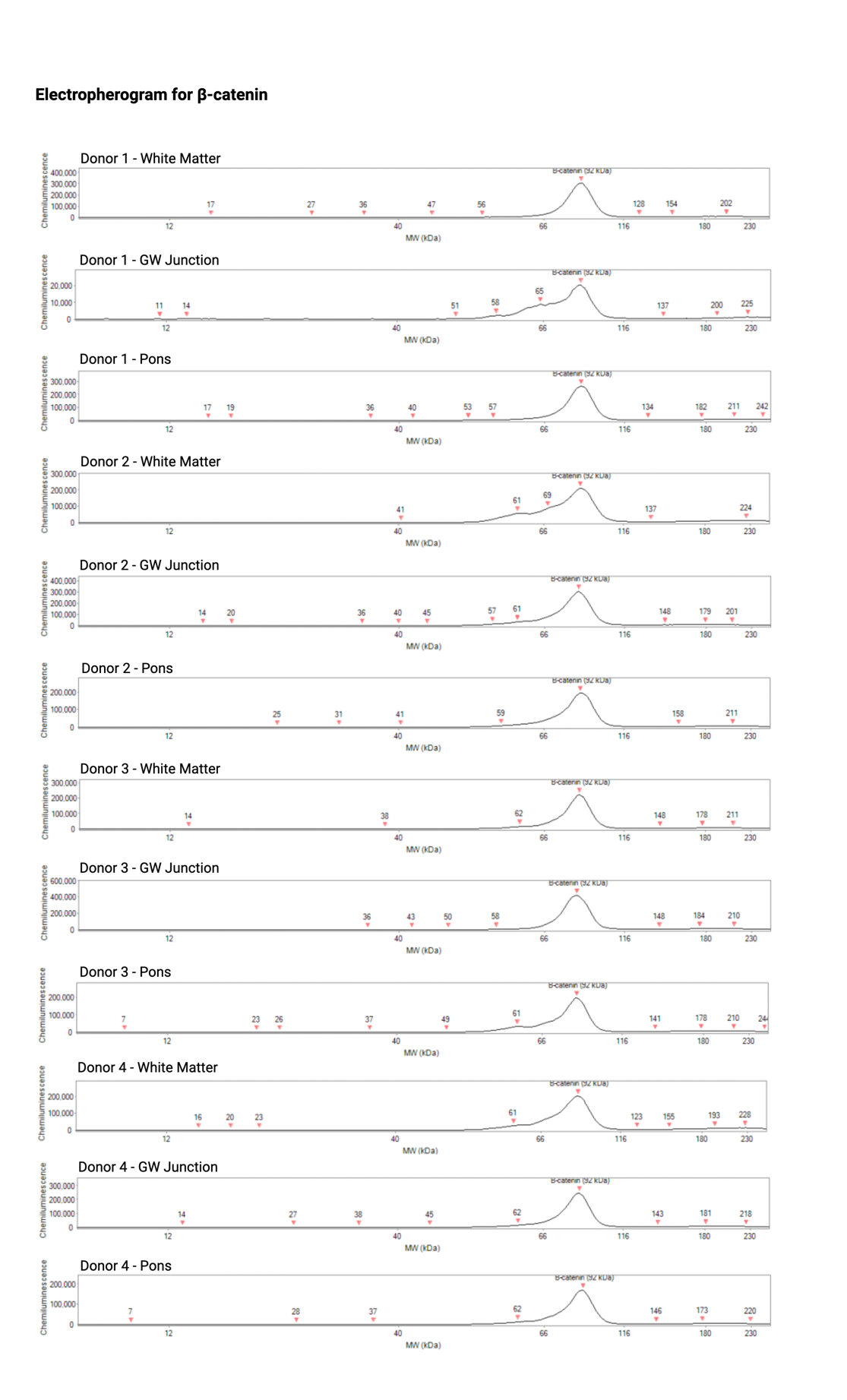

**Fig. S5** Electropherogram of capillary electrophoresis for $\beta$-catenin protein across brain regions (WM, GW Junction, and Pons) from different donors. Electropherograms corresponding to Fig. 2D showing chemiluminescence versus molecular weight (MW) indicating signal of bound proteins to the capillaries in the form of peaks on the graphs. $\beta$-catenin has a molecular weight of ~92kDa and is captured as such by its respective antibody on the electropherogram. Each donor and the respective brain regions have a signal peak for $\beta$-catenin at 92kDa.

**Tables**

Supplemental Table 1: Signal to Noise Ratios (S/N) for Immunoblotting Data

| **Donor # - Brain region** | **Primary Ab** | **Protien Name (Size kDa)** | **MW (kDa)** | **Area Under Curve (AUC)** | **S/N** |
| --- | --- | --- | --- | --- | --- |
| Donor 1- White Matter | Piezo1 | Piezo1 (286 kDa) | 275 | 92835.3 | 159.5 |
| Donor 1 - GW Junction | Piezo1 | Piezo1 (286 kDa) | 280 | 208409.5 | 55.9 |
| Donor 1- Pons | Piezo1 | Piezo1 (286 kDa) | 277 | 843390.6 | 129 |
| Donor 2- White Matter | Piezo1 | Piezo1 (286 kDa) | 276 | 253465.7 | 230.7 |
| Donor 2 - GW Junction | Piezo1 | Piezo1 (286 kDa) | 277 | 651937.2 | 185.6 |
| Donor 2 - Pons | Piezo1 | Piezo1 (286 kDa) | 276 | 714662.6 | 113.4 |
| Donor 3 - White Matter | Piezo1 | Piezo1 (286 kDa) | 274 | 522657.3 | 191.6 |
| Donor 3 - GW Junction | Piezo1 | Piezo1 (286 kDa) | 270 | 832425.5 | 110 |
| Donor 3 - Pons | Piezo1 | Piezo1 (286 kDa) | 276 | 932820.2 | 164.4 |
| Donor 4 - White Matter | Piezo1 | Piezo1 (286 kDa) | 280 | 422913 | 108.5 |
| Donor 4 - GW Junction | Piezo1 | Piezo1 (286 kDa) | 274 | 1191082.9 | 142.3 |
| Donor 4- Pons | Piezo1 | Piezo1 (286 kDa) | 270 | 905879.9 | 135.2 |
|  |  |  |  |  |  |
| Donor 1- White Matter | YAP | YAP (78 kDa) | 80 | 24932.3 | 294.2 |
| Donor 1 - GW Junction | YAP | YAP (78 kDa) | 80 | 18584.3 | 258.1 |
| Donor 1 - Pons | YAP | YAP (78 kDa) | 80 | 20811.2 | 226.6 |
| Donor 2 - White Matter | YAP | YAP (78 kDa) | 79 | 1831.3 | 21.8 |
| Donor 2 - GW Junction | YAP | YAP (78 kDa) | 77 | 20190.4 | 236 |
| Donor 2 - Pons | YAP | YAP (78 kDa) | 79 | 3353.4 | 32.7 |
| Donor 3 - White Matter | YAP | YAP (78 kDa) | 78 | 21112.3 | 222.6 |
| Donor 3 - GW Junction | YAP | YAP (78 kDa) | 78 | 78415 | 788.7 |
| Donor 3 - Pons | YAP | YAP (78 kDa) | 78 | 5781.4 | 75.8 |
| Donor 4 - White Matter | YAP | YAP (78 kDa) | 76 | 9164.9 | 90.2 |
| Donor 4 - GW Junction | YAP | YAP (78 kDa) | 76 | 5515.1 | 51.4 |
| Donor 4- Pons | YAP | YAP (78 kDa) | 79 | 5402.1 | 45.4 |
|  |  |  |  |  |  |
| Donor 1 - White Matter | PYAP | PYAP (78kDa) | 82 | 20417 | 130 |
| Donor 1 - GW Junction | PYAP | PYAP (78kDa) | 81 | 24697.3 | 158.2 |
| Donor 1 - Pons | PYAP | PYAP (78kDa) | 81 | 20960.8 | 184.7 |
| Donor 2 - White Matter | PYAP | PYAP (78kDa) | 78 | 957 | 9.5 |
| Donor 2 - GW Junction | PYAP | PYAP (78kDa) | 80 | 5980.3 | 57.3 |
| Donor 2 - Pons | PYAP | PYAP (78kDa) | 78 | 983 | 4.5 |
| Donor 3 - White Matter | PYAP | PYAP (78kDa) | 83 | 1929.8 | 24.3 |
| Donor 3 - GW Junction | PYAP | PYAP (78kDa) | 76 | 4654.1 | 32.4 |
| Donor 3 - Pons | PYAP | PYAP (78kDa) | 85 | 3213.7 | 37.1 |
| Donor 4 - White Matter | PYAP | PYAP (78kDa) | 80 | 3327.9 | 36 |
| Donor 4 - GW Junction | PYAP | PYAP (78kDa) | 85 | 2385.5 | 19.8 |
| Donor 4 - Pons | PYAP | PYAP (78kDa) | 83 | 2798.1 | 13.9 |
|  |  |  |  |  |  |
| Donor 1 - White Matter | B-catenin | B-catenin (92 kDa) | 89 | 4576240.1 | 24065.1 |
| Donor 1 - GW Junction | B-catenin | B-catenin (92 kDa) | 89 | 366817.2 | 4151.9 |
| Donor 1 - Pons | B-catenin | B-catenin (92 kDa) | 89 | 3929447.7 | 24729.3 |
| Donor 2 - White Matter | B-catenin | B-catenin (92 kDa) | 89 | 3355464.4 | 15456.3 |
| Donor 2 - GW Junction | B-catenin | B-catenin (92 kDa) | 87 | 4550107.7 | 34044.4 |
| Donor 2 - Pons | B-catenin | B-catenin (92 kDa) | 89 | 2935296.5 | 18712 |
| Donor 3 - White Matter | B-catenin | B-catenin (92 kDa) | 88 | 3082392.8 | 16385 |
| Donor 3 - GW Junction | B-catenin | B-catenin (92 kDa) | 86 | 6521933.6 | 24169.4 |
| Donor 3 - Pons | B-catenin | B-catenin (92 kDa) | 87 | 2789370.7 | 15540.4 |
| Donor 4 White Matter | B-catenin | B-catenin (92 kDa) | 89 | 3142938.5 | 14503.6 |
| Donor 4 - GW Junction | B-catenin | B-catenin (92 kDa) | 88 | 3656976.5 | 26431.1 |
| Donor 4 - Pons | B-catenin | B-catenin (92 kDa) | 90 | 2521621 | 8459.2 |
|  |  |  |  |  |  |
| Donor 1 - White Matter | B-actin | B-actin (48 kDa) | 48 | 1078172 | 1556.1 |
| Donor 1 - GW Junction | B-actin | B-actin (48 kDa) | 48 | 669275 | 698 |
| Donor 1 - Pons | B-actin | B-actin (48 kDa) | 48 | 803276.2 | 1779.6 |
| Donor 2 - White Matter | B-actin | B-actin (48 kDa) | 48 | 1166843 | 2331.7 |
| Donor 2 - GW Junction | B-actin | B-actin (48 kDa) | 48 | 1681955.7 | 3125.6 |
| Donor 2 - Pons | B-actin | B-actin (48 kDa) | 48 | 659823.1 | 1200.4 |
| Donor 3 - White Matter | B-actin | B-actin (48 kDa) | 48 | 1108416.6 | 1187.4 |
| Donor 3 - GW Junction | B-actin | B-actin (48 kDa) | 48 | 1048337.5 | 3449.9 |
| Donor 3 - Pons | B-actin | B-actin (48 kDa) | 48 | 742185 | 1805.4 |
| Donor 4 White Matter | B-actin | B-actin (48 kDa) | 48 | 1693631.4 | 2309.9 |
| Donor 4 - GW Junction | B-actin | B-actin (48 kDa) | 48 | 873992.8 | 2292.1 |
| Donor 4 - Pons | B-actin | B-actin (48 kDa) | 48 | 911018.8 | 1545.9 |

**Supplemental Table 1:** This table illustrates the signal-to-noise ratio attributed to each peak representing a protein band in Fig. 2 A-C. Signal-to-noise ratios >10 are considered true signals[1] for proteins of interest in addition to being validated based on size relative to the protein standard ladder present in the blot. Note that S/N ratio <10 may represent real signals however due to low endogenous protein expression in the brain certain proteins like pYAP may not consistently yield a S/N ratio >10.

**References:**

[1] P. Desharnais, J.-F. Naud, and C. Ayotte, “Detection of erythropoiesis stimulating agents in urine samples using a capillary Western system,” *Drug Testing and Analysis*, vol. 10, no. 11–12, pp. 1698–1707, 2018, doi: 10.1002/dta.2528.
